# Editorial: Immunometabolism: exploring the nexus of metabolism and immune function in health and disease

**DOI:** 10.3389/fimmu.2026.1827639

**Published:** 2026-03-19

**Authors:** Eric Henrique Roma, Juliana Lauar Gonçalves

**Affiliations:** 1 Laboratório de Imunologia e Imunogenética em Doenças Infecciosas, Instituto Nacional de Infectologia Evandro Chagas, Fundação Oswaldo Cruz, Rio de Janeiro, Brazil; 2 Centro Universitário UNA, Belo Horizonte, Brazil

**Keywords:** immune response, immunometabolic regulation, immunometabolism, infectious diseases, inflammation, non-infectious chronic diseases

Immunometabolism examines the relationship between metabolic processes and immune system function, providing deeper understanding of immunity. It is now established that the metabolic state of an immune cell is a key determinant of its activation, differentiation, and function (1). This relationship is bidirectional with not only the metabolism driving immune cell fate, but inflammatory signals also can induce profound metabolic shifts within these cells. This knowledge changed the view of the metabolism as only a housekeeping process to recognize it as an active regulator of immunity.

The interaction between metabolism and immunity can be observed in the immune cells’ functions. For example, upon activation, effector T cells undergo a metabolic switch from catabolic pathways, such as fatty acid oxidation (FAO), to anabolic pathways dominated by aerobic glycolysis, a process known as Warburg effect. This switch provides biosynthetic precursors (nucleotides, amino acids, and lipids) to support rapid cell proliferation and production of effector molecules like cytokines. Different T cell subsets are metabolically distinct: pro-inflammatory T helper 1 (Th1) and Th17 cells are highly glycolytic, whereas regulatory T cells (Tregs) rely on FAO and oxidative phosphorylation (OXPHOS) to sustain their function. Similarly, macrophages exhibit metabolic plasticity, with pro-inflammatory M1 macrophages favoring glycolysis, while anti-inflammatory M2 macrophages utilize FAO and OXPHOS (2).

In addition, metabolic dysregulation has direct consequences for immune function. In obesity and type 2 diabetes, excess nutrients create chronic, low-grade inflammation, or “meta-inflammation”. This environment impairs immune responses, increasing susceptibility to infections and promoting comorbidities. In the tumor microenvironment, high competition for nutrients between cancer cells and immune cells leads to T cell exhaustion and dysfunction, limiting anti-tumor immunity. These examples shows that the metabolic landscape is critical in shaping immune responses in both health and disease, opening new directions for therapeutic intervention.

Recent work explores molecular mechanisms linking metabolism and immunity across disease contexts. At the level of fundamental mechanisms, epitranscriptomics has emerged as a key regulatory layer. Wang et al. provides an overview of how N6-methyladenosine (m6A) RNA modification regulates macrophage metabolic reprogramming. The authors detail the roles of m6A “writers” (METTL3), “erasers” (FTO, ALKBH5), and “readers” (YTHDF proteins), which modulate the stability and translation of key metabolic and inflammatory transcripts. m6A modification can promote expression of genes involved in glycolysis, facilitating M1 macrophage polarization. This work points to the therapeutic potential of targeting m6A pathways to modulate macrophage function in metabolic diseases (3, 4).

Metabolites themselves also act as signaling molecules. Tada and Kono mini-review focuses on immunometabolites that directly regulate immune cell activity in autoimmune diseases such as rheumatoid arthritis and systemic lupus erythematosus. The authors describe the context-dependent role of lactate, which can be pro-inflammatory by promoting IL-17 production in Th17 cells but also immunosuppressive by inhibiting T cell proliferation. They highlight the anti-inflammatory functions of itaconate, a TCA cycle-derived metabolite that inhibits succinate dehydrogenase, reducing mitochondrial reactive oxygen species (ROS) production and suppressing the NLRP3 inflammasome. These findings suggest that targeting enzymes responsible for producing or degrading these metabolites could be a viable therapeutic strategy.

In oncology, the review by Moretti et al. adopts a systemic perspective, considering how host metabolism influences cancer progression and treatment response. The authors discuss how metabolic conditions like obesity and cachexia induce systemic inflammation and alter nutrient availability, impairing T cell function and promoting immune evasion. They address the “obesity paradox,” where obesity has been associated with improved outcomes for checkpoint inhibitor therapy in some cancers, potentially due to hyper-activated T cells in the obese state. The review also explores cholesterol metabolism, noting that while cancer cells require cholesterol for proliferation, high cholesterol levels can induce T cell exhaustion through immune checkpoint molecule expression. An original research article by Gómez de Cedrón et al. presents findings from a randomized, double-blind clinical trial evaluating a bioactive formula containing diterpenic phenols from rosemary (Lipchronic^©^) in cancer patients. The formula reduced systemic inflammation markers, such as the neutrophil-to-lymphocyte ratio (NLR), particularly in the lung cancer subgroup. This suggests nutritional interventions targeting immunometabolic pathways may serve as a complementary cancer management strategy.

In infectious diseases, Kanno et al. reviewed the complex interplay between host metabolism, immune response, and viral pathogens. The authors summarize how viruses reprogram host cell metabolism to create favorable environments for replication, often by hijacking glucose and lipid pathways (5, 6). Many viruses enhance glycolysis to provide ATP and building blocks for virion production. The review discusses how these metabolic alterations influence the host immune response. A key focus is type I interferon (IFN-I) signaling, which not only has direct antiviral effects but also enhances fatty acid oxidation (FAO) and oxidative phosphorylation (OXPHOS) in immune cells like plasmacytoid dendritic cells, required for their optimal function (7). This highlights a metabolic tug-of-war between virus and host immune system.

Finally, the connection between systemic metabolism, inflammation, and nutrition is explored in an original research article by Liao et al. The authors analyze data from the National Health and Nutrition Examination Survey (NHANES) to investigate the relationship between the systemic immune-inflammation index (SII)—calculated from neutrophil, platelet, and lymphocyte counts—and visceral adipose tissue (VAT) area. The study found a positive correlation between SII and VAT, particularly in overweight and obese individuals. These findings provide evidence for obesity as chronic low-grade inflammation and reinforce the link between visceral adiposity and systemic immune activation (8).

Together, the contributions offer a multifaceted exploration of immunometabolism, highlighting emerging mechanisms and therapeutic targets shaping the field. From epitranscriptomic regulation of macrophage function (Wang et al.) and metabolite signaling in autoimmunity (Tada and Kono), to systemic metabolic effects on cancer (Moretti et al.; Gómez de Cedrón et al.), viral pathogenesis (Kanno et al.), and obesity-driven inflammation (Liao et al.), these articles collectively contribute to our understanding of how energy metabolism intersects with health and disease. They point toward innovative strategies for diagnosis, prevention, and treatment across human conditions. We hope this Research topic stimulates further research and dialogue in the field of immunometabolism.
